# A Gender-Affirming Approach to Fertility Care for Transgender and Gender-Diverse Patients

**DOI:** 10.1097/og9.0000000000000002

**Published:** 2024-03-19

**Authors:** William J. Powers, Dustin Costescu, Carys Massarella, Jenna Gale, Sukhbir S. Singh

**Affiliations:** Powers Family Medicine, Farmington Hills, Michigan; and McMaster University, Hamilton, and the University of Ottawa, Ottawa, Ontario, Canada.

## Abstract

Although many transgender and gender-diverse (TGD) people express a desire to become parents, providing quality counseling that encompasses patients' short-term and long-term fertility options often represents a challenge for health care professionals. Fertility care (from preservation to restoration or assisted reproduction or both) has a low uptake in the TGD population, with barriers including financial cost, concerns regarding health care professionals’ biases, and patient misconceptions or lack of awareness of fertility outcomes. Fertility care is particularly important for the TGD population, because gender-affirming hormone therapy can negatively affect fertility. Despite this importance, existing guidelines and research on this subject are limited. This article summarizes the currently available fertility care options for TGD patients and provides our clinical opinion on their best provision. We highlight that it is essential for health care professionals to provide TGD patients with holistic counseling to facilitate informed decision making about their reproductive health before initiating gender-affirming therapy. Fertility counseling should outline the options available for the patient, keeping their needs and preferences at the center of the conversation while advising on the associated risks and financial costs.

## Abstract 2

We highlight the need for more research, guidelines, and sharing of clinical experiences on fertility care for transgender and gender-diverse patients.

Many transgender and gender-diverse (TGD; see definitions in Table 1) people express a desire to become parents.^1,2^ Despite this, *fertility care*, which may include fertility preservation, fertility restoration, assisted reproduction, or a combination of these, is an afterthought for many health care professionals serving TGD patients.

**Table 1.** Definitions of Terms Related to Transgender Health Care*

Term	Definition
Assigned female at birth (AFAB)	A person, irrespective of gender identity, whose sex assignment at birth resulted in a declaration of “female” (including transgender men)
Assigned male at birth (AMAB)	A person, irrespective of gender identity, whose sex assignment at birth resulted in a declaration of “male” (including transgender women)
Cisgender, cis	A person whose gender identity corresponds with that of their sex assigned at birth
Gender-affirming	Behaviors or interventions that affirm a person's gender identity
Gender dysphoria	A sense of psychological discomfort or distress that a person may feel as a result of disparity between their gender identity and their sex assigned at birth
Gender identity	The personal sense of one's own gender
Preferred gender pronouns	The set of pronouns that a person uses and wishes others to use to reflect their gender identity; these may include gender-neutral pronouns (such as they and them) or neopronouns (such as ze and zir)
Transgender, trans	A person whose gender identity does not correspond with that of their sex assigned at birth
Transgender and gender-diverse (TGD)	A person whose gender identity does not correspond with that of their sex assigned at birth, including those with gender identities that lie outside the gender continuum, between the binary gender categories of male and female, or as a combination of male and female genders
Transgender woman, trans woman	A person whose gender identity is female but whose sex assigned at birth was male
Transgender man, trans man	A person whose gender identity is male but whose sex assigned at birth was female

* This list is not exhaustive; there are many terms used to describe and express gender identity.

Gender-affirming therapy (including hormonal or surgical treatment or both) is an important component of gender transitioning for many TGD patients. However, these treatments may affect patients' short-term and long-term fertility.^3–5^ For example, testosterone treatment in individuals assigned female at birth (AFAB; see definitions in Table 1) and estrogen treatment in individuals assigned male at birth (AMAB; see definitions in Table 1) may lead to reduced fertility.^6^ The extent to which the effects of gender-affirming hormone therapy (GAHT; see definitions in Table 1) are reversible is currently unknown. Additionally, most surgical methods of gender affirmation, such as hysterectomy–oophorectomy in AFAB individuals and orchiectomy in AMAB individuals, are permanent.

International guidelines recommend that health care professionals discuss fertility care and reproductive aspirations with patients before they initiate GAHT or undergo gender-affirming surgery.^7–10^ Unfortunately, these guidelines often lack best-practice recommendations for fertility counseling, and conversations in standard care often overlook fertility preservation for TGD individuals as a result.^11,12^

The uptake of fertility preservation treatments in the TGD population is low, particularly among adolescents.^13–15^ In contrast to historical practices, local laws and regulations in most regions no longer prevent the TGD community from accessing fertility care. Ethics committees are also fully supporting the provision of fertility services for TGD individuals.^10^ Yet, barriers to accessing and using fertility care remain. These include a lack of insurance coverage, the high costs associated with fertility care, and concerns around being misgendered or mistreated by health care professionals.^1,14–16^ One study showed that transgender patients and their partners had largely negative interactions with health care professionals when accessing or attempting to access fertility care.^17^ In the broader context of health care, TGD patients face an absence of trans-friendly environments. Examples include the unwillingness or lack of understanding from health care professionals in acknowledging gender identities through the use of gender-affirming language and preferred names and pronouns (see definitions in Table 1).^18,19^

It is also important to consider that TGD patients may be worried about the effects of fertility care interventions on their gender transitions. Possible concerns include delays to the start of GAHT, unwillingness to cease GAHT, and potentially triggering or exacerbating gender dysphoria (see definitions in Table 1).^1,13,20^ Effective counseling from health care professionals may help to alleviate some of these concerns and increase fertility care uptake.

Health care professionals often feel uncomfortable or unconfident in providing fertility care to TGD patients due to the limited education and guidance on this topic.^16,20,21^ Although training for health care professionals is improving, one study on urology and plastic surgery programs reported that the median training duration dedicated to transgender care was approximately 1 didactic hour and 2 clinical hours.^22^ Another study showed that only approximately 50% of surveyed health care professionals had received any form of training in transgender care, despite more than 90% believing that such training would increase their competence.^23^

Seeing that gender-affirming therapies could hinder the ability to have children, the scarcity of health care professional guidance and consequently low uptake of fertility preservation represent a major concern for many TGD individuals who desire parenthood.^1,2^ This article reviews the current guidance and makes additional recommendations for managing fertility care for TGD patients, with the aim of addressing the paucity of guidance in the literature.

## FERTILITY CARE FOR AFAB PATIENTS

For AFAB individuals, testosterone therapy is the predominant GAHT.^7,8^ The long-term effects of testosterone on fertility are uncertain. Previous studies have suggested that long-term testosterone treatment may lead to endometrial atrophy by impairing endometrial growth and function^24^ or induce polycystic ovarian morphology.^25,26^ Other studies have found that testosterone therapy has no effect on follicular distribution or ovarian architecture and does not induce transitions to polycystic ovarian morphologies.^27,28^ A recent retrospective cohort study reported similar fertilization rates and preimplantation embryo development between cisgender patients (see definitions in Table 1) and testosterone-treated AFAB patients who had ceased testosterone therapy for 3 months before fertility preservation.^29^

In addition to GAHT, AFAB patients may also undergo gender-affirming surgery, which involves oophorectomy, hysterectomy, salpingectomy, or a combination of these.^7,8,30^ Because these surgeries might permanently alter fertility, careful counseling in respect to fertility implications is mandatory before a procedure. For example, a patient posthysterectomy who retains their ovaries has the potential to have genetically related offspring (carried by their partner, if their partner is AFAB, or a gestational carrier), whereas a patient who retains their uterus after oophorectomy would need an oocyte source (or previously cryopreserved oocytes), despite being able to carry a pregnancy. A patient who underwent salpingectomy could have a pregnancy only through in vitro fertilization (IVF).^30^

### Fertility Preservation

To preserve fertility, AFAB patients may opt to forgo gender-affirming treatment until after they have finished childbearing. If the patient wishes to begin gender-affirming therapy before attempting pregnancy, they may undergo a fertility-preservation procedure. The clinically available options for fertility preservation in AFAB patients are embryo, oocyte, and ovarian tissue cryopreservation (although the latter is not widely available).^7,8^ These options can be performed before, during, or after initiating GAHT.

Controlled ovarian hyperstimulation, commonly achieved by prescribing recombinant gonadotropins (eg, follicle-stimulating hormone and luteinizing hormone), is often used to facilitate embryo and oocyte cryopreservation.^31,32^ Controlled ovarian hyperstimulation usually is an effective procedure for improving fertility outcomes in AFAB TGD patients. After ovarian stimulation and oocyte retrieval, AFAB TGD individuals with a history of GAHT typically see fertility outcomes similar to those of cisgender women or AFAB TGD individuals who have not undergone GAHT.^6,31,32^

Ovarian tissue cryopreservation is another method of fertility preservation. During gender-affirming surgery, ovarian tissue can be removed, frozen, and stored. This tissue then can be reimplanted at a later date when the patient desires pregnancy.^3–5,33^ Of note, the patient should cease GAHT before reimplantation to avoid inhibiting the function of the ovarian tissue.^3–5,33^ In addition, immature oocytes can be sourced from ovarian tissue to undergo an in vitro maturation process at a later stage. This procedure is experimental but promising, particularly for patients who do not wish to cease GAHT and those for whom fertility was not restored after ovarian tissue reimplantation.^28,34^

It is important to consider that these fertility treatments all could risk triggering patients' gender dysphoria, because they often involve surgeries, procedures, hormone therapies, or a combination of these that are specific to the sex with which the patient no longer identifies. Clinicians, therefore, should manage these treatments sensitively, with continued attention to patients' needs and desires. For example, although transvaginal ultrasonography is necessary to monitor ovarian stimulation, transgender patients must be well-counseled before the procedure to alleviate concerns with vaginal examination. Moreover, there are additional factors that limit real-world access to advanced reproductive technologies, including local regulation, availability of specialist health care professionals, and technological and financial constraints.

As is the case with elective oocyte cryopreservation in age-related fertility decline, the age at which oocytes are cryopreserved and the ovarian reserve of the patient can strongly influence the chance of a live birth.^35^ Ideal outcomes occur most often in patients younger than age 35 years who have normal or high ovarian reserve.^35^ It is important to note that a successful birth is not guaranteed, even under favorable conditions.

### Increasing and Restoring Fertility

Fertility restoration is often not necessary for AFAB patients who have been undergoing GAHT. In the past, the common belief was that AFAB TGD patients seeking fertility preservation must cease GAHT until their periods resume, which can be distressing and lead to gender dysphoria. However, recent evidence has shown that prolonged cessation of GAHT may not be necessary. One case report suggests that testosterone needs to be discontinued only immediately before and during ovarian stimulation.^36^ Two further studies have supported the feasibility of cryopreserving mature oocytes without the need for AFAB patients to cease their long-term testosterone GAHT.^37,38^ Additionally, a published case series reports that not all patients were able to resume menses after ceasing testosterone GAHT for 2–3 months to undergo oocyte cryopreservation.^32^ Ceasing GAHT may, therefore, do more harm than good, because its potential to trigger gender dysphoria outweighs the limited benefits to fertility.

That being said, fertility restoration still can be beneficial for some patients, such as those looking to achieve natural pregnancy and restore ovulation as quickly as possible, as well as those hoping to undergo oocyte retrieval. In Box 1, we present protocols used in our clinical practice for increasing fertility in AFAB patients.

Box 1. **Clinical Opinion: Increasing Fertility in Patients Who Were Assigned Female at Birth**

In AFAB individuals hoping to achieve natural pregnancy and restore ovulation as quickly as possible, or for those hoping to undergo oocyte retrieval, we recommend the following protocol:

- If the patient is undergoing oocyte retrieval, they may first discontinue masculinizing GAHT to maximize regimen success (although this may not be essential).
- The cessation of GAHT is essential if the patient desires pregnancy due to the teratogenic effects of testosterone.
  ○ Testosterone should be ceased for a minimum of 5 half-lives before attempting pregnancy (eg, testosterone cypionate with a half-life of 7–10 d should be ceased for at least 50 d).
- If menstruation occurs within 30 d, no intervention is needed for restoration of ovulation.
- If menstruation does not occur within 30 d, a progesterone challenge test can be performed to induce bleeding (eg, medroxyprogesterone acetate 10 mg taken orally daily for 5–10 d).
- If natural pregnancy or IUI is desired and menses has not resumed, ovulation induction then can be performed using a SERM (eg, oral clomiphene 50 mg or oral raloxifene 60 mg taken daily on days 3–7 of the menstrual cycle) or an aromatase inhibitor (eg, oral letrozole 2.5 mg taken daily on days 3–7 of the menstrual cycle).

• It should be noted that SERMs have both antiestrogenic and estrogenic effects and so will cause breast atrophy (although this is generally reversible).^52^
• A control ultrasonogram should be performed on day 8 of the menstrual cycle.
• Ovulation induction can be repeated monthly, with dosage increases if ovulation is not successful within a given cycle, until oocyte retrieval or pregnancy occurs.

- For individuals desiring oocyte retrieval for cryopreservation or IVF, we recommend following standard IVF protocols to achieve ovarian stimulation (eg, FSH or LH or both injected daily, with a starting dose of 150–200 international units/d).

This regimen is an off-license approach for transgender AFAB patients due to the limited research and guidelines surrounding transgender fertility care. However, this method has documented success and is a standard practice among cisgender women.^53,54^

AFAB, assigned female at birth; GAHT, gender-affirming hormone therapy; IUI; intrauterine insemination; SERM, selective estrogen receptor modulator; IVF, in vitro fertilization; FSH, follicle-stimulating hormone; LH, luteinizing hormone.

### Assisted Reproduction

Assisted reproduction is an option for AFAB patients, although the methods used are dependent on the fertility-affecting procedures that the patient has undergone previously (such as hysterectomy, which would preclude the patient from carrying a pregnancy). The requirements for successful pregnancy also depend on the patient's partner (or donor).

Intrauterine insemination (IUI) and IVF are techniques that use the sperm from a patient's AMAB partner or donor to fertilize oocytes from the AFAB patient. Intracytoplasmic sperm injection (ICSI) can be used to improve the chances of success with IVF, where indicated.^5,39^ At this stage, embryos may be cryopreserved if desired. A gestational carrier may be necessary to carry the pregnancy to term if the AFAB patient has already undergone gender-affirming surgery or does not wish to carry the pregnancy themselves.^3–5^

Reciprocal IVF, a process in which the partner carries the pregnancy, is an option for AFAB patients whose partner is also AFAB. In this case, however, a sperm donor will be required for fertility treatment, IVF, or IUI. The resultant embryo may then be transferred into the patient's uterus, the partner's uterus, or into the uterus of a gestational carrier.^3,5^

It is critical to note that, if an AFAB individual will be carrying the pregnancy, they must cease GAHT completely before trying for pregnancy due to the teratogenic effects of testosterone.^21^ Because pregnancy can greatly trigger gender dysphoria, thorough counseling is essential for patients considering pregnancy. Special considerations for mode of delivery may be necessary if, for example, vaginal examinations and procedures are not possible.

**Fig. 1.**
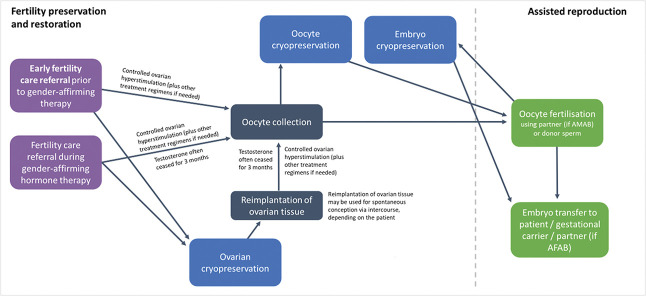
Fertility options for patients assigned female at birth (AFAB). AMAB, assigned male at birth.

A summary of the fertility options for AFAB patients is presented in Figure 1.

*Powers. Gender-Affirming, Transgender Fertility Care. Obstet Gynecol 2024*.

## FERTILITY CARE FOR AMAB PATIENTS

Feminizing GAHT for AMAB patients usually takes the form of estrogens and androgen-reducing medications.^7,8^ Feminizing GAHT inhibits spermatogenesis and reduces sperm motility and sperm count, but the long-term effects and reversibility of this type of therapy are unknown.^38,39^ Some AMAB individuals may also choose to undergo orchiectomy as a form of gender-affirming surgery.^7,8^ Because orchiectomy is an irreversible procedure, fertility preservation must be carried out before it.

### Fertility Preservation

If a patient desires parenthood, they may wish to delay their gender-affirming therapy until after they have had children. This is particularly important for AMAB patients due to the lack of evidence for fertility restoration after estrogen therapy.^3,5,8,38,39^ Early referral for fertility care is, therefore, crucial for the AMAB TGD population.

Currently, sperm cryopreservation is the only clinically available fertility-preservation option for AMAB individuals.^3–5^ Assessment of sperm viability and sperm cryopreservation ideally should be carried out before the initiation of GAHT to maximize the possibility of collecting high-quality sperm. In cases in which the patient has initiated GAHT already, and no sperm is present within the ejaculate, it is recommended to pause GAHT for at least 3 months to allow spermatogenesis to occur and sperm count to increase. The presence of sperm in the ejaculate then may be confirmed before cryopreservation to improve the efficacy of this procedure.^5^

Feminizing GAHT can lead to problems with sperm quantity and quality, erections, and ejaculation.^3,5,8,40,41^ Sperm collection through masturbation also can trigger gender dysphoria in some patients.^42^ In this case, surgical sperm retrieval, through which sperm is taken directly from parts of the testis or epididymis, can be a viable alternative.^43^ This may be performed at the same time as gender-affirming surgery to limit the number of surgeries for the patient.

Although fertility preservation for AMAB patients has become increasingly simple and accessible, the cost of sperm cryopreservation and storage still presents a significant barrier. This is particularly true for the TGD community, because they are a marginalized group at higher risk for financial difficulties.^5,15^

### Fertility Restoration

Fertility restoration for AMAB patients should be used only when fertility preservation has not been carried out or was unsuccessful. There is a lack of evidence supporting the viability of fertility restoration after the initiation of GAHT in AMAB TGD patients. However, small studies have found that therapeutic effects on fertility may be reversible if treatment is ceased even for a period as short as 2 weeks.^40,41^ Another study examining cryopreserved semen quality in AMAB TGD individuals shows that the quality of samples collected after discontinuing GAHT was comparable with those collected from patients who had not undergone GAHT. On the other hand, current users of GAHT had lower semen quality than patients who had not undergone GAHT.^42^

Conflicting evidence also exists; other studies have shown that cryopreserved semen samples from AMAB TGD patients, regardless of GAHT history, were of lower quality than those from matched cisgender individuals in a control group.^44,45^ Additionally, a larger study by de Nie et al found significantly lower semen quality among AMAB TGD individuals without a history of GAHT when compared with World Health Organization data for the general population.^46^ It should be noted, however, that the authors observed no correlation between semen quality and GAHT in AMAB patients who had ceased GAHT for at least 3 months.

In the same study, only approximately 25% of cryopreserved and subsequently warmed samples from TGD patients had semen quality sufficient for IUI.^46^ Known risk factors—including obesity, alcohol consumption, cannabis use, and smoking—were insufficient to explain this low quality. The authors postulated that psychological stress, depression, and anxiety may contribute to decreased quality.^46^ Moreover, lifestyle factors, such as the practice of keeping the genitals tight against the body or pushing the testicles into the inguinal canal (tucking), might increase scrotal temperatures and reduce semen quality.^46,47^ The cryopreservation of low-quality semen often results in even lower quality after warming and, consequently, precludes the success of IUI.

Taken together, existing literature on semen quality highlights the importance of early fertility preservation and the shortfalls of fertility restoration in AMAB patients. Of note, certain protocols have seen some success despite the aforementioned limitations. These are presented in Box 2.

Box 2. **Clinical Opinion: Fertility Restoration for Patients Who Were Assigned Male at Birth**

When treating a currently azoospermic AMAB patient wishing to restore their fertility, it is important for the health care professional and the patient to be aware that the duration of normal spermatogenesis is 74 d. Thus, it will take a minimum of 74 d after ceasing GAHT for semen samples to become viable. It is, therefore, recommended to wait 90 d after the patient has ceased GAHT before taking the first sperm sample.

There currently is no research supporting the use of medical therapies to restore fertility in the transgender AMAB population. Protocols for this population have been extrapolated from off-label therapies used in hypogonadal cisgender males.^55–57^ One such protocol that may be effective in restoring fertility in AMAB patients is detailed below:

- SERMs are used to induce the recovery of normal testicular function (eg, oral clomiphene 50 mg taken daily in a 30-d cycle of 25 d on and 5 d off, or oral raloxifene 60 mg taken using the same regimen).
  ○ It is important to note that this will, in turn, cause a surge in testosterone levels, which may be very dysphoric and unpleasant for patients.
  ○ It should be noted that SERMs have both antiestrogenic and estrogenic effects, and so will cause breast atrophy (although this is generally reversible).^52^
- After 90 d of SERM treatment, blood samples can be drawn to measure LH, FSH, and testosterone levels, and a semen sample can be collected to determine sperm viability.
  ○ If LH and FSH levels are elevated beyond the normal range, this indicates that the regimen is producing a physiologic response in the patient. Elevations in testosterone then indicate that the testes are responding to the elevation in LH and FSH levels, thereby demonstrating a response to the regimen.
- If sperm is not yet viable, the SERM protocol can be continued for a further 90 d, after which hormone levels and sperm viability should be remeasured.
- If sperm still is not viable after 180 d, the SERM protocol may be continued with the addition of hCG (eg, injected as 2,000 international units every 3 d).

Because guidelines for transgender fertility care are limited and use of protocols is off-label, clinicians should use their clinical judgment when deciding on treatment regimens for their patients. It is important to note that there is no guarantee that sperm viability will be restored; if it is, it may not return to pre-GAHT levels. Thus, early referral for fertility preservation is key in the AMAB transgender population.

AMAB, assigned male at birth; SERM, selective estrogen receptor modulator; LH, luteinizing hormone; FSH, follicle-stimulating hormone; hCG, human chorionic gonadotropin; GAHT, gender-affirming hormone therapy.

### Assisted Reproduction

The pregnancy requirements of AMAB individuals vary depending on their partner or donor. If their partner is also AMAB (or if the patient wishes or needs to use donor sperm), a donor oocyte and a gestational carrier will be required to carry the embryo to term.^3–5^ If the patient has an AFAB partner, IUI may be carried out as long as the sperm is of sufficient quality.^3–5^ Although IUI is minimally invasive and less expensive than IVF, it often requires the use of a considerable amount of semen. This is because pregnancy rates after IUI are generally low, at approximately 10–20% per cycle, and higher amounts of semen cryopreservation often are needed to maximize the chance of success.^48^ Therefore, it would be advisable to cryopreserve multiple semen samples (which confers significant financial costs) if the patient would like to attempt IUI.

In vitro fertilization and ICSI are techniques generally used to increase the chance of pregnancy, especially for patients who have a low sperm count or low-quality sperm. However, they are both more invasive and expensive.^3,5^ Barring these concerns, IVF often is recommended even in cases in which the quality and quantity of sperm are sufficient, because it confers higher pregnancy and live-birth rates per cycle than IUI.^5,38,49^ For patients with particularly poor-quality semen (low motility and low sperm count), ICSI should be recommended to improve the chances of fertilization.^5,38,49^

**Fig. 2.**
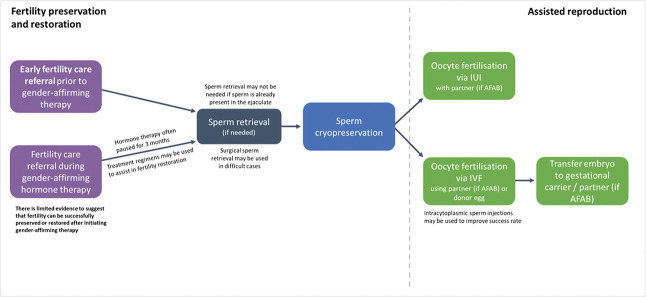
Fertility options for patients assigned male at birth (AMAB). AFAB, assigned female at birth; IUI, intrauterine insemination; IVF, in vitro fertilization.

A summary of the fertility options for AMAB patients is presented in Figure 2.

*Powers. Gender-Affirming, Transgender Fertility Care. Obstet Gynecol 2024*.

## REFLECTIONS ON DISCUSSING FERTILITY CARE WITH TGD PATIENTS

It is essential to provide TGD patients with fertility care counseling so that they can make informed decisions about their reproductive health, ideally before initiating fertility-affecting GAHT. Less experienced health care professionals often feel difficulty when providing quality fertility care for TGD patients. As such, we have created a script that could serve as guidance for these conversations (Box 3).

Box 3. **Tips for Patient Counseling**

Having a script to follow when counseling transgender patients about fertility care can be useful. This can be adapted and individualized depending on the patient.

Below is an example of a script that may be used to guide fertility care and ensure that all key areas are covered.

For AFAB patients:

- Discuss with your patient their wishes and desires around starting a family in the future.
- Inform your patient that the effect of long-term testosterone GAHT on oocyte quality and quantity is largely unknown.
  ○ There have been many successful reports of oocyte cryopreservation and pregnancy after starting testosterone GAHT (eg, in cases in which testosterone was stopped 2–3 months before oocyte cryopreservation as recommended, or in some rare circumstances in which testosterone was continued up to or throughout ovarian stimulation and oocyte retrieval).
  ○ Oophorectomy would preclude a genetic child, and preservation would need to be considered before surgery.
  ○ Ovarian tissue cryopreservation, although not widely available, may be an option, particularly for patients who are considering oophorectomy.
- Review with your patient that undergoing a fertility evaluation and fertility preservation, including an oocyte retrieval, involve the use of transvaginal ultrasonography. Transvaginal ultrasonography can cause significant gender dysphoria and may be painful or uncomfortable, especially if the patient has not previously had vaginal penetration (whether through intercourse or a prior pelvic examination or tampon use) or has pain with vaginal penetration.
- Discuss oocyte cryopreservation (freezing eggs to preserve fertility) with your patient in the context of GAHT. This option is key if the patient considers having a genetically related child important.
  ○ The most important predictors of success are the patient's age and ovarian reserve at the time that oocytes are cryopreserved.
  ○ Evaluation of the patient's ovarian reserve and the accessibility of their ovaries for oocyte retrieval is completed by transvaginal ultrasonography and a blood test (AMH level).
- Review the many options for starting a family in the future.
  ○ Fostering and adoption.
  ○ Fertilization using cryopreserved oocytes and partner or donor sperm, after which the embryo is transferred into your patient's uterus, the uterus of a partner, or the uterus of a gestational carrier.
  ○ Fertilization using donor oocytes and partner or donor sperm, after which the embryo is transferred into your patient's uterus, the uterus of a partner, or the uterus of a gestational carrier.
  ○ Seeking pregnancy before starting or after stopping testosterone therapy using a partner's sperm (intercourse) or donor sperm (IUI or IVF).
- Review IVF
  ○ Whether using an oocyte from your patient or a donor oocyte, pregnancy rates are primarily dependent on the age of the oocyte provider when the oocyte was cryopreserved, with optimal pregnancy rates seen using oocytes cryopreserved when the oocyte provider was younger than age 35 years.
  ○ After the embryo has formed, it can be transferred into the uterus of your patient, their partner, or a gestational carrier.

For AMAB patients

- Discuss with your patient their wishes and desires for starting a family in the future.
- Discuss the option of fertility preservation using sperm cryopreservation.
  ○ Sperm cryopreservation should take place before initiating GAHT or gender-affirming surgery if having a genetically related child is important to your patient.
  ○ Estrogen GAHT can have a significant and negative effect on spermatogenesis (creation of new sperm) and has the potential to irreversibly affect fertility.
  ○ The recovery of sperm is variable and unpredictable after ceasing estrogen GAHT. The ejaculate of many patients undergoing estrogen GAHT does not contain sperm, and the chance of this being reversed (restoring fertility) after ceasing estrogen GAHT (with or without hormone treatment to boost sperm production) is unknown. There is a significant risk that sperm retrieval would not be possible.
- Review the process of sperm cryopreservation.
  ○ Sperm ejaculation is retrieved. The ejaculated sperm is processed and cryopreserved.
  ○ Cryopreserved gametes typically are used only in IVF with ICSI, given that a significant proportion of the sperm does not survive the cryopreservation and subsequent warming process.
  ○ To be able to use cryopreserved sperm for insemination in the future, multiple cryopreservation samples with sufficient precryopreservation semen parameters typically are required to achieve reasonable pregnancy rates.
- Review the process of IVF.
  ○ Whether using an oocyte from your patient's partner or a donor oocyte, pregnancy rates are primarily dependent on the age of the oocyte provider when the oocyte was cryopreserved, with optimal pregnancy rates seen using oocytes cryopreserved when the oocyte provider was younger than age 35 years.
  ○ After the embryo has formed, it can be transferred into the uterus of your patient's partner or a gestational carrier.

For all patients

- Review the potential negative effects and risks associated with fertility-care procedures (eg, gender dysphoria, risks associated with invasive procedures, failure and negative outcomes, fetal or maternal complications).
- Advise your patient about the financial costs of these procedures.
- Advise your patient about where to seek further advice and support if needed (eg, from mental health specialists, transgender support groups, financial support groups).
- Set up follow-up appointments with your patient to maintain a continued presence and open dialogue.

It is important to counsel your patient in an individually tailored manner, keeping them and their needs and desires at the center of the discussion.

AFAB, assigned female at birth; GAHT, gender-affirming hormone therapy; AMH, anti-müllerian hormone; IUI, intrauterine insemination; IVF, in vitro fertilization; AMAB, assigned male at birth; ICSI, intracytoplasmic sperm injection.

Building trust and rapport is vital.^50^ Health care professionals should demonstrate an awareness of the trauma and discrimination experienced by TGD patients and use gender-affirming language. A recent survey of transgender patients in Canada reported key characteristics of health care professionals that promote positive health care experiences.^51^ These include health care professionals who are knowledgeable, experienced, and willing to learn about transgender health; health care professionals who empower and center patients in health care discussions; and health care professionals who are sensitive, accepting, and validating of patients' gender identities.^51^ It is also important not to make assumptions about the patient, such as their gender identity or sexual orientation (see definitions in Table 1). Multidisciplinary teams, including but not limited to pediatricians (if the patient is an adolescent), obstetricians, gynecologists, reproductive endocrinologists, infertility specialists, and financial advisors, may help to establish a network of specialists and build a community of support.^12^

Counseling should outline the options available for TGD patients while keeping their needs and preferences at the forefront of discussions. Along with fertility preservation, restoration, and assisted reproduction, health care professionals should discuss other options for achieving parenthood, for example, adoption, donor gametes, and surrogacy. Health care professionals also should note whether the patient has already received any gender-affirming treatment, because this will influence the most suitable methods of fertility care. If the patient desires to have children or undergo fertility care in the near future, they may consider delaying or ceasing GAHT until after the fertility care is complete.

If the patient has a partner, they may wish to include their partner in fertility-care counseling; indeed, this may be a requirement in some jurisdictions. Pregnancy requirements (eg, surrogacy, sperm donation) should be thoroughly explained to both individuals, especially because these may differ depending on whether the patient and their partner are AMAB or AFAB. More importantly, fertility procedures come with risks associated with the invasiveness of procedures, failure and negative outcomes, fetal or maternal complications, and gender dysphoria. Health care professionals always should advise patients on these risks and the financial costs associated with different procedures. Counseling should be ongoing, holistic, and adaptable to provide support tailored to the patient's situation, preferences, and wishes over time.

## CONCLUSION

As health care professionals, we should strive to ensure that all our TGD patients have their fertility needs met in a gender-affirming manner. Despite the importance of fertility care in this population, there is currently a lack of research into the effect of GAHT on fertility. Only limited guidelines give suggestions on how to best counsel and care for TGD patients. The sharing of clinical opinions on TGD fertility care allows for the exchange of valuable ideas, provides guidance where little is available, and encourages research to improve holistic care in this population.
